# Cerebellar and Spinal Direct Current Stimulation in Children: Computational Modeling of the Induced Electric Field

**DOI:** 10.3389/fnhum.2016.00522

**Published:** 2016-10-17

**Authors:** Serena Fiocchi, Paolo Ravazzani, Alberto Priori, Marta Parazzini

**Affiliations:** ^1^Istituto di Elettronica e di Ingegneria dell’Informazione e delle Telecomunicazioni (IEIIT), Consiglio Nazionale delle Ricerche (CNR)Milan, Italy; ^2^Dipartimento di Scienze della Salute, Ospedale San Paolo, Università degli Studi di MilanoMilan, Italy

**Keywords:** ctDCS, tsDCS, computational modeling, neuromodulation, high-resolution human models, children

## Abstract

Recent studies have shown that the specific application of transcranial direct current stimulation (tDCS) over the cerebellum can modulate cerebellar activity. In parallel, transcutaneous spinal DC stimulation (tsDCS) was found to be able to modulate conduction along the spinal cord and spinal cord functions. Of particular interest is the possible use of these techniques in pediatric age, since many pathologies and injuries, which affect the cerebellar cortex as well as spinal cord circuits, are diffuse in adults as well as in children. Up to now, experimental studies of cerebellar and spinal DC stimulation on children are completely missing and therefore there is a lack of information about the safety of this technique as well as the appropriate dose to be used during the treatment. Therefore, the knowledge of electric quantities induced into the cerebellum and over the spinal cord during cerebellar tDCS and tsDCS, respectively, is required. This work attempts to address this issue by estimating through computational techniques, the electric field distributions induced in the target tissues during the two stimulation techniques applied to different models of children of various ages and gender. In detail, we used four voxel child models, aged between 5- and 8-years. Results revealed that, despite inter-individual differences, the cerebellum is the structure mainly involved by cerebellar tDCS, whereas the electric field generated by tsDCS can reach the spinal cord also in children. Moreover, it was found that there is a considerable spread toward the anterior area of the cerebellum and the brainstem region for cerebellar tDCS and in the spinal nerve for spinal direct current stimulation. Our study therefore predicts that the electric field spreads in complex patterns that strongly depend on individual anatomy, thus giving further insight into safety issues and informing data for pediatric investigations of these stimulation techniques.

## Introduction

In the last decade two innovative techniques, cerebellar transcranial direct current stimulation (ctDCS) and transcutaneous spinal direct current stimulation (tsDCS), based on the delivery of direct current transcutaneously, have been proven to affect and modulate the neural activity in the cerebellum and in the spinal cord, thus offering promising therapeutic opportunities for restoring their functions (Priori et al., 2014).

It is known that weak electrical currents can induce persisting excitability changes in the stimulated structure (Woods et al., 2016). In detail, ctDCS can modify cerebello-brain networks, affect locomotion and motor learning skills, enhance cognitive functions and also improve the treatment of cerebellar disorders (see the review studies of Priori et al., 2014; Ferrucci et al., 2015; Grimaldi et al., 2016). In parallel, given the existing spinal-brain interactions and the need to find a non-invasive neuromodulatory tool to prevent neuronal dysfunctions developed after spinal cord injuries, the possibility to apply transcutaneous direct current over spinal cord was explored. This gave significant insights that tsDCS can effectively modulate conduction along the spinal somatosensory pathways and alter spinal cord functions (for a review see Cogiamanian et al., 2012; Priori et al., 2014).

Of particular interest is the possible application of these techniques in pediatric age, since many pathologies and injuries, which affect the cerebellar cortex as well as the spinal cord circuits, are diffuse in adults as well as in children. So far, experimental studies on tDCS failed to report side effects in adults (Nitsche et al., 2003; Poreisz et al., 2007; Brunoni et al., 2011), but no systematic experimental data are available in children. Indeed, up till now, very few studies have applied tDCS in pediatric population (Schneider and Hopp, 2011; Varga et al., 2011; Yook et al., 2011; Siniatchkin et al., 2012; Gillick et al., 2015; Moliadze et al., 2015), mainly using the classical tDCS montages with both electrodes on the scalp (i.e., the one with the active electrode over the motor cortex-M1 and the reference electrode over the contralateral supraorbital cortex).

However, it should be considered that, because the intensity of the current generated in the neural tissues during stimulation depend both on the tDCS dose (montage and current intensity) and the interposed tissues architecture, the same dose applied to an adult is expected to produce different current flows in the neural tissues of children and adolescents and hence could have critical implications for tDCS safety and efficacy (Bikson et al., 2012). Indeed, lately, Kessler et al. (2013) have shown by a modeling study, a better electric current transmission effectiveness in the brain of children than of adults, mainly due to the increase in scalp-brain distances with age and the consequent increases in skull thickness and extra-axial cerebrospinal fluid (CSF) space.

Moreover, in a recent modeling study of cerebellar tDCS on adults and adolescents (Parazzini et al., 2014b), the authors found a higher electric field spread towards the anterior area of the cerebellum in the adolescent model than in the adult models.

In light of these findings, a link between the individual anatomical variability and the spread of the electric quantities can be argued. Similarly, in Parazzini et al.’s (2014a) study, where the field distributions over the spinal cord of adult and adolescent models were assessed, the role of the individual anatomical variability was confirmed also for tsDCS. However, these findings both on ctDCS and tsDCS should be verified by enlarging the analysis to other models of variable ages.

This work aims, therefore, to better explore this issue, using pediatric magnetic resonance imaging (MRI)-derived whole-body anatomical models of children of different ages and gender. With the purpose to gain significant and quantitative observations useful for the application of pediatric tDCS, we in particular, estimated through computational electromagnetics techniques, the electric field distributions induced in the cerebellum, in the brain and in the brainstem during cerebellar tDCS and in the spinal cord and spinal nerve roots during tsDCS.

By providing a quantitative estimate of the electric field distributions induced in the neural structures, this work could be of some help in understanding the relationship between the setting parameters of cerebellar and spinal direct current stimulation and the resulting current flow in the target tissues, thus providing the possibility to advance anatomy-based dose design considerations.

## Materials and Methods

### Human Models

We used four child models, aged between 5- and 8-years, of the Virtual Population (Christ et al., 2010) whose details and anthropometric quantities of interest are summarized in the following table (Table 1).

**Table 1:** **Virtual population models anatomical characteristics**.

	Roberta	Thelonious	Eartha	Dizzy
Age (years)	5	6	8	8
Gender	F	M	F	M
Height (m)	1.10	1.15	1.36	1.37
Weight (kg)	17.8	19.3	30.7	26.0
BMI (kg/m^2^)	14.9	14.1	16.6	13.8
**Cerebellum**
(Max) Antero-posterior length (cm)	6.6	6.8	6.8	7.0
CSF volume (cm^3^)	30.7	34.6	48.6	88.6
(Mean) Skull thickness (mm)	8.4	8.1	10.9	8.7
**Spinal chord**
CSF volume (cm^3^)
*Cervical*	4.8	9.5	14.5	10.6
*Thoracic*	16.2	12.2	21.4	20.3
*Lumbar*	7.4	6.1	44.8	17.2
Volume (cm^3^)
*Cervical*	4.6	6.9	7.1	5.9
*Thoracic*	6.6	16.8	6.2	7.5
*Lumbar*	3.3	2.3	1.1	5.9
Length (cm)
*Cervical*	7.3	8.7	10.0	10.6
*Thoracic*	17.0	17.0	17.9	21.4
*Lumbar*	10.8	11.4	13.0	14.5

Their different ages allows exploring the potential application of both the DC stimulation techniques over most of childhood. The models derive from the high-resolution segmentation of magnetic resonance (MR) images of healthy volunteers and an accurate computer-aided design representation of the organ surfaces. The segmentation allows distinguishing up to 76 different tissues in the whole body, with some limitations due to problems associated with MRI acquisition sequences and reconstruction algorithm, which result in shading effects and artifacts. This is, for example, the case of the CSF volume in the cerebellar region where in all the models, but with variability across them, there are some points where the thickness of the CSF, filling the space between the brain and the skull, is lower than the grid resolution, leading to a direct contact between the brain and the skull. This results in a variable volume of the CSF itself at the level of the cerebellum across the models.

**Figure 1:**
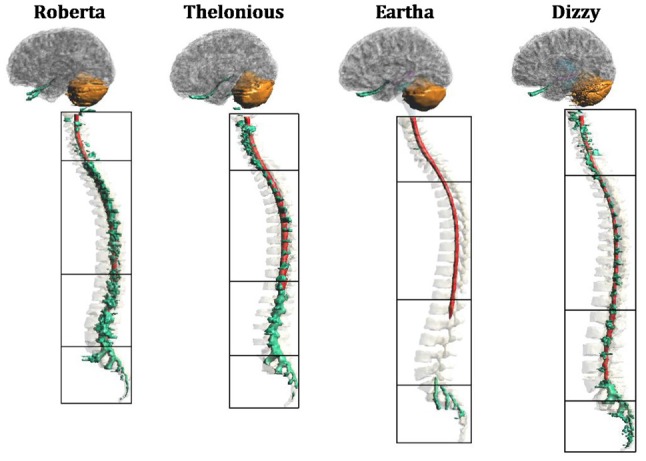
**Segmentation masks for (from left to right) “Roberta”, “Thelonious”, “Eartha” and “Dizzy”.** Lateral view of cerebellum (orange), spinal cord (red) and spinal nerves (green) with vertebrae and cerebral tissues in transparency. Black boxes distinguish the four levels of the vertebrae (cervical, thoracic, lumbar and sacral).

Figure 1 depicts the segmentation masks of the spinal cord, spinal nerve roots and cerebellum, i.e., the main regions of interest of the present study, for each child model. One can note that the cauda equina is only partially segmented in Roberta, and not segmented in the other three models. Moreover, at lumbar level, Roberta’s spine is interrupted whereas Thelonious and Eartha’s spines are only partially segmented in their upper part. Similarly, the spinal nerve roots are well segmented along the spine of all models but Eartha, has a good representation of nerve roots only in the upper part of the sacral segment. Consequently, in the discussion of the results, these approximations have to be taken into account.

**Figure 2:**
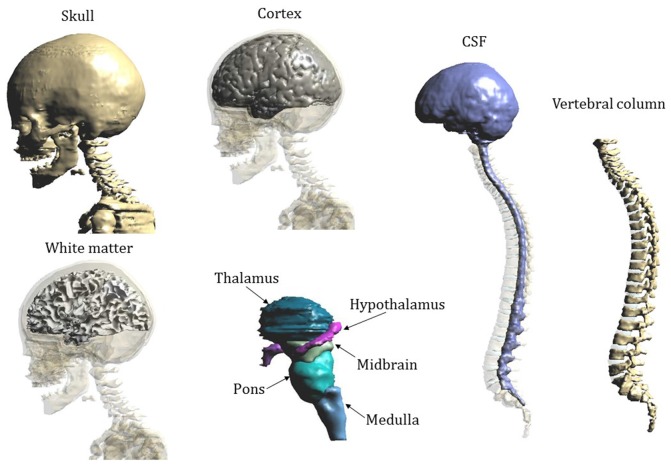
**Segmentation masks of Eartha’s tissues of interest close to the cerebellum and the spinal cord**.

Figure 2 shows the segmentation masks of other tissues of interest close to the cerebellum and the spinal cord for model “Eartha”.

Tissues’ dielectric properties at DC are limited to very few tissues (Miklavčič et al., 2006). Since our human models instead contain a large number of tissues, we assigned the dielectric properties of any other extra tissue according to data at low frequency (i.e., 10 Hz) collected in the comprehensive Gabriel study (Gabriel et al., 1996; Gabriel, 1997), following an approach already described in other tDCS modeling studies (e.g., Parazzini et al., 2014a,b). Table 2 summarizes the conductivity values assigned to the different tissues.

**Table 2:** **Tissues conductivity**.

Tissue	Conductivity (S/m)
Adrenal gland, epididymis, esophagus, hypophysis, pancreas, pineal body, small intestine, small intestine lumen, stomach, stomach lumen, thymus thyroid gland	0.511
Air internal, bronchi lumen, pharynx, trachea lumen	0
Artery, blood vessels, hearth lumen, penis, vein	0.7
Bladder	0.203
Bone, mandible, marrow red, skull, teeth, vertebrae	0.0200
Brain gray matter, hippocampus, hypothalamus, thalamus	0.0275
Brain white matter, commissura anterior, commissura posterior	0.0277
Breast	0.262
Bronchi, ureter-urethra	0.251
Cartilage, ear cartilage, intervertebral disks, larynx, trachea	0.161
Cerebellum	0.0475
Cerebro spinal fluid (CSF)	2
Connective tissue	0.122
Cornea, prostate, testis	0.411
Diaphragm, muscle	0.202
Ear skin, skin	0.1
Eye lens, ovary	0.311
Eye sclera	0.501
Eye vitreous humor	1.5
Fat, Subcutaneous adipose tissue (SAT)	0.0122
Gallbladder	0.9
Hearth muscle	0.0537
Kidney cortex, kidney medulla	0.0544
Large intestine, large intestine lumen, vagina	0.0122
Liver	0.0277
Lung	0.121
Medulla oblongata, midbrain, pons	0.0276
Mucosa	0.0004
Nerve, spinal cord	0.0171
Spleen	0.0396
Tendon ligament	0.251
Tongue	0.261
Uterus	0.201

### Electrodes Modeling

**Figure 3:**
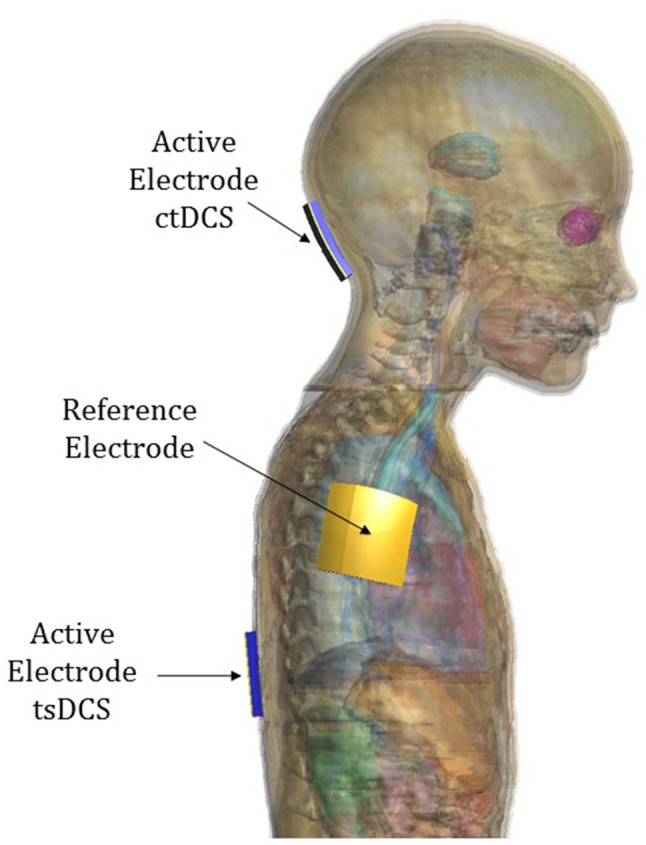
**Electrode positioning over model “Eartha”**.

The active electrode was placed on the scalp over the cerebellar area in ctDCS and over the spinous process of the 10th thoracic vertebra for tsDCS, whereas the reference electrode was placed over the right arm in both montages. The electrodes were modeled as rectangular pads conductors (*σ* = 5.9 × 10^7^ S/m) of 1 mm of thickness placed above a rectangular sponge (*σ* = 0.3 S/m), of the same size of the electrodes with a thickness of 5 mm (Figure 3). Their dimensions and positions are summarized in the following table (Table 3).

**Table 3:** **Dimension and position of the two electrodes in the two montages of cerebellar transcranial direct current stimulation (ctDCS) and transcutaneous spinal direct current stimulation (tsDCS)**.

	ctDCS		tsDCS	
	Dimension	Position	Dimension	Position
Active electrode	5 × 5 cm^2^	Over cerebellum, centered over the median line 2 cm below the inion	5 × 3 cm^2^	Centered over the spinal process of the 10th thoracic vertebra
Reference electrode	5 × 5 cm^2^	Right arm	5 × 5 cm^2^	Right arm

### Numerical Simulations

A simulation-based approach as implemented by the simulation platform SEMCAD X (by SPEAG, Schmid and Partner Engineering, AG, Zurich, Switzerland^1^) was used to compute the electric field distributions. The Laplace equation was solved to determine first the electric potential (ϕ) distribution:

(1) ∇·(σ∇φ) = 0

where σ (S/m) is the electrical conductivity of the human tissues. The electric field (**E**) distributions were obtained by means of the following relations:

(2) E = -∇φ

According to the maximum current tested in clinical studies, the potential difference between the electrodes was adjusted to inject a total current of 2 mA for cerebellar tDCS and 3 mA for tsDCS, as done in the previous modeling studies (Parazzini et al., 2014a,b).

For each simulation, the human model and the electrodes were inserted in a surrounding bounding box filled with air, and all the models were truncated at the shoulder level for cerebellar tDCS and at the thigh level for spinal tDCS. At the bounding box face corresponding to the truncation section has been assigned the boundary conditions of continuity of the current, whereas the others faces of the bounding box are treated as insulated i.e., the normal component of the current density was set equal to zero. Continuity of the tangential component of **E** was applied at each tissue-to-tissue boundary. At the interface between the skin and the air, the current density was set to be parallel to the face. Uniform rectilinear meshes were applied to discretize the computational domain with a grid discretization step ranging from 0.5 mm to 0.7 mm. Those dimensions were allowed to finely discretize even structures, such as the spinal cord, which have a tiny dimension, and in the meantime to determine the results with a reasonable computational cost. The resulting meshes range from 325.27 million to 457.08 million mesh cells for ctDCS and from 320.94 million to 394.82 million mesh cells for tsDCS simulations.

### Electric Field Characterization

According to the recommendations provided by the International Commission on Non-Ionizing Radiation Protection (2010) the induced **E** was calculated as a vector average of the **E** in a small contiguous tissue volume of 2 mm^3^ × 2 mm^3^ × 2 mm^3^, as a practical compromise satisfying both requirements for a flawless biological basis and computational limits.

Therefore, in the following section, the results will be presented in terms of this definition and the **E** distributions will be analyzed on the target tissues of each stimulating technique. Specifically, they include cerebellum, occipital cortex, white matter and deeper structures (pons, midbrain, medulla, thalamus, hypothalamus and hippocampus) in cerebellar tDCS and spinal cord, cauda equina and spinal nerves in transcutaneous spinal tsDCS.

In particular, the **E** distribution in each cerebral structure of interest and in spinal cord and nerves at the different spine levels will be described in terms of quartiles, minimum and 99th percentile. The last percentile was chosen instead of the maximum to filter spurious points due to the staircase errors. For the sake of brevity, in the following section we will use always the word “maximum” or “peak” for the 99th percentile of the distribution.

In the ctDCS simulation, we quantified the spread of the field towards other brain structures than the cerebellum as the percentage volume of these structures where the **E** amplitude was greater than the 70% of the maximum of **E** amplitude in the cerebellum. Moreover, the spread of **E** within the cerebellum was analyzed in terms of percentage of volume of the cerebellum that is exposed to an **E** amplitude equal to or greater than the 50% and the 70% of its maximum (V50 and V70, respectively), following the approach proposed by Parazzini et al. (2014b).

Similarly, in the tsDCS simulations, for the characterization of the uniformity of the **E** distributions on transversal sections along the spine, we calculated the coefficient of variation (CV; i.e., the ratio between the standard deviation and the mean) of the **E** amplitude distribution in different tracts along the spine for all the models. Moreover, given the directional effects of **E** in the interaction with neurons (Rushton, 1927; Rattay, 1986; Roth, 1994), we also analyzed the directional behavior of **E** distribution along the spine. This was done by calculating the mean of the ratio (R) evaluated slice by slice along the vertebral column between the longitudinal and transversal (i.e., the root square of the quadratic sum of the dorsoventral and mediolateral components) field components at different spine levels for all the human models, following the approach proposed by Parazzini et al. (2014a).

## Results

### ctDCS Electric Field Distribution

**Figure 4:**
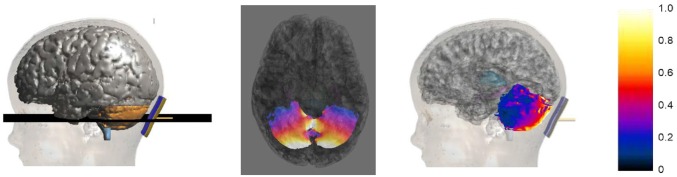
**Axial section across the cerebellum (2nd column) of the **E** amplitude distribution for Roberta.** The first column on the left shows the plane where the section was calculated. The third column shows the sagittal view of the **E** amplitude distribution over the cerebellar surface. The colored scale on the right is normalized with respect to the maximum of the **E** amplitude in the cerebellum.

Figure 4 shows an example of the **E** amplitude distribution on an axial slice passing through the cerebellum (2nd column) and on the cerebellar surface (3rd column) for the model Roberta. The values of the color map are normalized with respect to the maximum of the **E** amplitude in the cerebellum.

The distributions clearly show that the strongest electric field is induced mainly near the active electrode in the posterior lobe of the cerebellum, with some spread toward the anterior parts.

**Figure 5:**
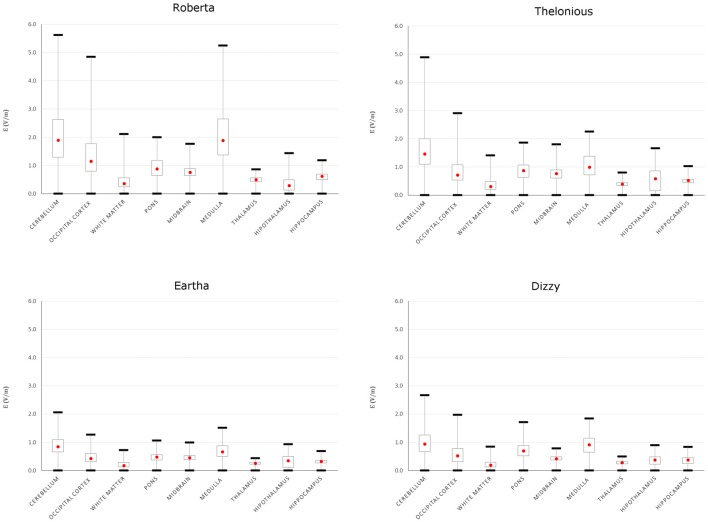
**Descriptive statistic of **E** amplitude over cerebellum and close brain tissues of the four children models.** The boxes indicate the interquartile range (25th–75th), red point the median (or 50th) value and the whiskers the minimum and maximum (or 99th) values.

To better evaluate the levels of **E** amplitude, Figure 5 shows the descriptive statistic, in terms of minimum, maximum, 25th, 50th and 75th percentiles of the** E** distribution induced over the cerebellum and other brain tissues for each model. The strongest electric field occurred in the cerebellum, with both peak and median levels showing a decreasing trend with age: the younger the child, the higher are the field levels induced in the cerebellum. The decrease with respect to the same values found in Roberta (i.e., the youngest child) ranged from 13.1% in Thelonious to 63.4% in Eartha for peak levels and from 23% in Thelonious to 55.6% in Eartha for median levels.

Besides the cerebellum, the electric field spreads toward other brain regions, in particular the occipital cortex and the medulla, with a minimum contraction of the peak respect to the value found in the cerebellum of about 13.7% and 6.7%, respectively, for the Roberta model. Interestingly, that peak decrease is much higher in the oldest models (i.e., Dizzy and Eartha) with a decreasing percentage of about 30–40%, but is maximum in the medulla of the 6-year old model (Thelonious), where it reaches up to 54%. The spread towards the occipital cortex and the medulla was also quantified as the percentage of volume of these structures where the amplitude of **E** is greater than 70% of the peak of **E** found in the cerebellum. It was found lower than the 1% in both tissues and in all the models, but Roberta, where it results in a maximum of 2% in the occipital cortex and reaches up to the 5% in the medulla.

The median levels over all the other brainstem and deep brain regions and across all the four child models stay under 1 V/m.

To better compute the spread within the cerebellum, Table 4 summarizes the percentage of volume of the cerebellum of each model, with an **E** amplitude higher than the 50% and 70% of its peak. Among the different models, Eartha shows a more widespread **E** amplitude distribution in the cerebellum, whereas Thelonious is characterized by the minimum spread of the distribution (around the half compared to Eartha) for both the metrics evaluated. An almost comparable spread in the distributions of Roberta and Dizzy within the cerebellum was found.

**Table 4:** **Percentage volume of the cerebellum with an **E** amplitude higher than the 50% and 70% of peak of each model**.

	Roberta	Thelonious	Eartha	Dizzy
V50 (%)	20.3	12.9	30.8	21.0
V70 (%)	6.4	4.4	8.9	6.0

**Figure 6:**
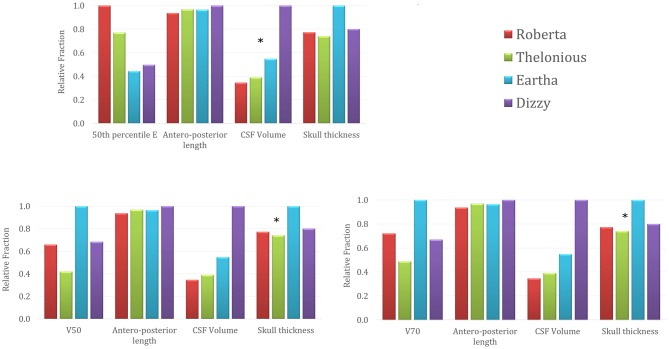
**50th percentile of the **E** amplitude in the cerebellum (top row), V50 (left) and V70 (right) percentages trends across the four models in comparison with the trends of three anthropometric quantities (i.e., maximum cerebellar antero-posterior length, cerebro spinal fluid (CSF) volume around cerebellum and maximum skull thickness in the occipital bone).** The stars identify the anthropometric quantities showing a similar trend to the respective electric field quantity.

Figure 6 represents the trend across the four models of the 50th percentile of the **E** amplitude distributions in the cerebellum (top row), the **E** amplitude spread percentages within the cerebellum (V50 in the bottom left and V70 in the bottom right) in comparison with the trend of three anthropometric quantities of interest for the cerebellar stimulation, i.e., the maximum antero-posterior length of the cerebellum, the CSF volume at the cerebellar level and the maximum skull thickness in the occipital bone (Table 1). For the sake of readability, the levels of each quantity are normalized with respect to the maximum value that it reaches in each model and they are represented as relative fraction of that maximum. Just from a visual inspection, one can notice, as an example, a lack of correlation between the trend of the 50th percentile of the **E** amplitude in the cerebellum and the antero-posterior length of the cerebellum, or the skull thickness at the cerebellar level. On the contrary, there is an evident negative dependence between the trends of the 50th percentile and the CSF volume at the cerebellar level, for all the models. Similarly, the pattern of V50 and V70 is similar to the maximum skull thickness in the occipital bone pattern.

### tsDCS Electric Field Distribution

**Figure 7:**
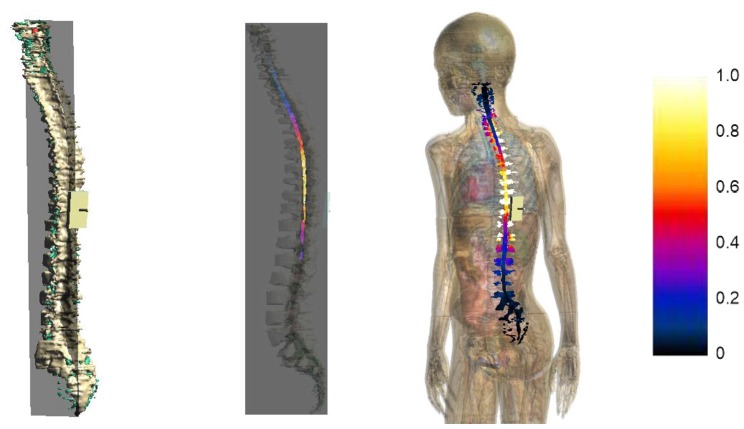
**Sagittal section across the spinal cord of the **E** amplitude distribution (2nd column) for Dizzy.** The first column on the left shows the plane where the section was calculated. The third column shows a view of the **E** amplitude distribution over the spinal cord and nerve surface. The color scale on the right is normalized with respect to the maximum of **E** amplitude in the spinal cord.

Figure 7 represents the **E** amplitude distributions on a sagittal slice passing through the spinal cord (2nd column) and on the spinal cord and nerve surface (3rd column) of model Dizzy, here taken as an example. The values of the color map are normalized to the maximum of **E** amplitude in the spinal cord.

The distributions clearly show that the strongest electric field occurred both in nerves and in the spinal cord, mainly near the active electrode at the thoracic level, with some spread toward the thoracic tract and the superior lumbar tract.

**Figure 8:**
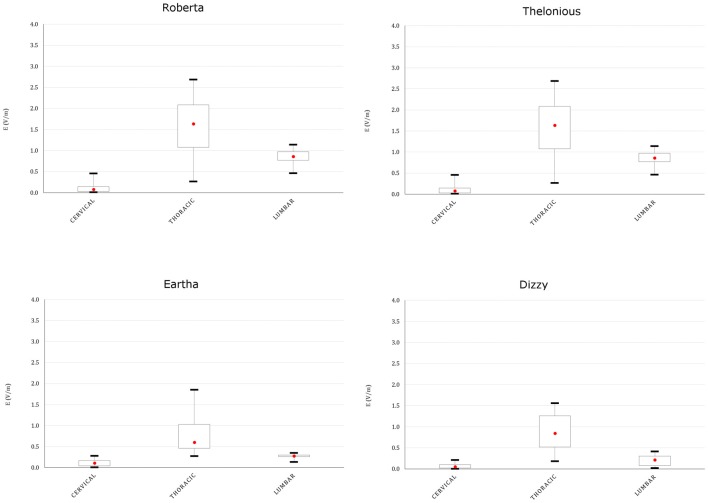
**Descriptive statistic of **E** amplitude distribution over the spinal cord at different spine levels, across the four models**.

**Figure 9:**
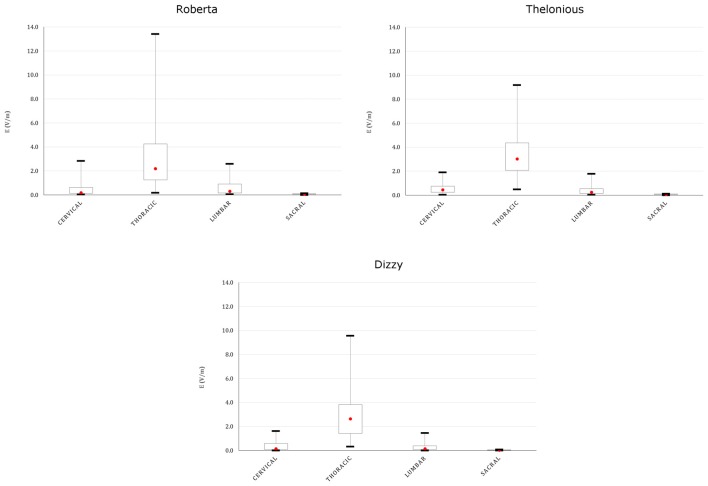
**Descriptive statistic of **E** amplitude distribution over the spinal nerves in different spinal nerve segments, across the three models whose nerves are represented (Eartha not shown)**.

This spread is more evident both on the youngest models’ spinal cord and nerves, as indicated in Figures 8, 9, showing the descriptive statistic of the **E** distributions induced over the spinal cord (Figure 8) and spinal nerves (Figure 9) across the models. The peaks at thoracic level in the spinal cord and nerves are highest in Thelonious and Roberta, respectively. On the contrary, comparable median levels over both the spinal cord and the nerves were obtained in all models. At cervical level and, if the pertinent structure is segmented, at sacral level, **E** amplitude substantially decreases more than 80% of the **E** amplitude at thoracic level in both nerves and spinal cord and in both median and peak levels and across all the models.

Table 5 summarizes the mean CV of **E** amplitude over the transverse sections of the spinal cord. The **E** amplitude distribution on transverse sections at the thoracic level is always more uniform (mean CV lower than 7.2%) than in the other levels, whereas the highest variations were found mainly in the cervical region.

**Table 5:** **Mean coefficient of variation (CV) of **E** amplitude distribution along the spinal cord calculated at the three different spinal levels**.

Mean CV spinal cord (%)	Roberta	Thelonious	Eartha	Dizzy
Cervical	8.1	11.5	8.7	12.4
Thoracic	6.7	7.2	6.7	7.0
Lumbar	5.0	4.8	7.2	6.4

**Figure 10:**
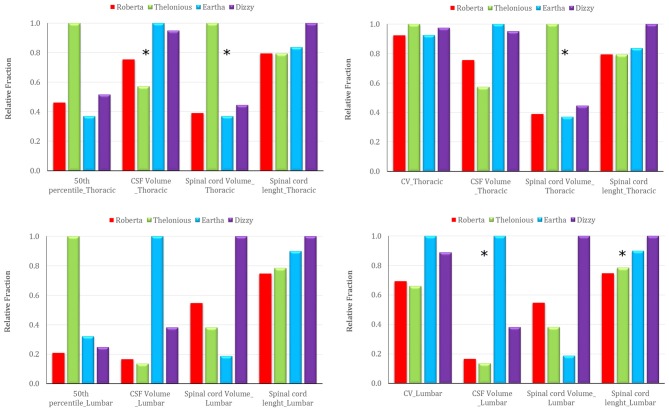
**Trend of the 50th percentile of the **E** amplitude distribution in the spinal cord (left), and of the CV over transverse section (right) across the four models in comparison with the trend of three anthropometric quantities at thoracic (top) and lumbar (down) level (i.e., CSF volume, spinal cord volume and spinal cord length).** The stars identify the anthropometric quantity that presents a similar trend to the respective electric field quantity.

Figure 10 reports the trend across the four models of the 50th percentile of the **E** amplitude distribution in the spinal cord (left column), and the CV over the transverse section (right column) in comparison with the trend of the three anthropometric quantities of interest for the spinal stimulation, i.e., the CSF volume, the spinal cord volume, the spinal cord length at the thoracic (top row) and lumbar level (bottom row; Table 1). A possible dependence results between the 50th percentile trend and the CSF volume (negative correlation) and spinal cord volume (positive correlation) trends at thoracic level, whereas a lack of dependence at lumbar level. Also, the CV trend presents a different behavior in the two central levels: it seems to be positively linked to the spinal cord volume at thoracic levels, and both to the CSF volume and to the spinal cord length at lumbar level.

The direction of **E** along the spine can be gleaned by Table 6, which shows the mean ratio between longitudinal and transverse **E** components calculated in the four spine regions for each model. As expected, **E** is mainly directed longitudinally in the regions closest to the electrode: at thoracic levels (mean R higher than 1.7) and at lumbar level (mean R higher than 1), whereas a prevalent direction can’t be identified at cervical level.

**Table 6:** **Mean of the ratio (R) evaluated between the longitudinal and transverse field components at different spine levels (cervical, thoracic and lumbar) for all the human models**.

Rmean	Roberta	Thelonious	Eartha	Dizzy
Cervical	1.24	0.86	0.53	1.13
Thoracic	1.71	1.87	2.99	4.08
Lumbar	1.03	2.03	1.30	3.88

### Cardiac Safety

The use of an extra-cephalic reference electrode could pose, for both the montages here investigated, some issues related to the cardiac safety (Vandermeeren et al., 2010; Parazzini et al., 2013). Table 7 reports the median and peak levels of **E** amplitude delivered directly to the heart during both modulation techniques.

**Table 7:** **Median (50th percentile) and peak levels of the **E** distribution over heart for both montages and for all the human models**.

E (V/m)		Roberta	Thelonious	Eartha	Dizzy
*ctDCS*	50th percentile	0.16	0.17	0.13	0.15
	Peak	0.41	0.50	0.46	0.41
*tsDCS*	50th percentile	1.28	0.97	0.82	0.85
	Peak	3.60	1.63	2.26	1.90

## Discussion and Conclusions

We here discussed the first computational modeling study on the electric field distributions induced by cerebellar and transcutaneous spinal tDCS (ctDCS and tsDCS, respectively) in children models.

This work completes the analysis started by two previous modeling studies conducted on adults (Parazzini et al., 2014a,b) and provides some indications about the electric field distribution that can give further insight into safety issues of pediatric tDCS investigations.

### ctDCS

In terms of **E** amplitude (Figure 4), both the maximum and median levels over the cerebellum of the two 8 year old models are equivalent or even lower than the ones found by the previous modeling study conducted on two adults and one adolescent (Parazzini et al., 2014b). Conversely, they are slightly higher in the two youngest models, thus suggesting that the dose settings have to be accurately discussed when this technique is applied in the youngest children. This is probably due to the overall neuroanatomic differences between children and adults that may contribute to the change in electric fields observed in these two studies.

However, despite inter-individual variability, the pattern of **E** amplitude distribution in the cerebellum (Figures 4, 5) and close tissues (Figure 5) is qualitatively comparable in the adult models, adolescents (Parazzini et al., 2014b) and among children; the cerebellum being the primarily involved structure by stimulation followed by the occipital cortex and medulla. The spread at 70% of the peak of cerebellum was found very slight in all the models, but in Roberta, it reached up to 5% (similar to the spread V70 within the cerebellum of Table 4) in the medulla. These findings are partially consistent with the previous experimental results showing that cerebellar tDCS at 2 mA failed to affect visual evoked potential (Ferrucci et al., 2008) and to alter brainstem excitability (Galea et al., 2009), but have to be better investigated in particular when the distance between the medulla and the active electrode is reduced, as in case of the 5 years old child (i.e., Roberta). We still do not know whether the possible brainstem spread is functionally relevant.

Still about the safety, the use of extra-cephalic reference electrode could pose some issues related to the cardiac electrophysiology. The average threshold for cardiac fibrillation converge to 5 A/m^2^ for large electrodes, value supported by data from experiments of both human and dog hearts (Reilly, 2012). Similarly to what postulated in our previous studies (Parazzini et al., 2013, 2014b), the peak levels found here, ranging from 0.41 V/m to 0.50 V/m in terms of **E** (Table 7) or from 0.022A/m^2^ to 0.025 A/m^2^, in terms of **J** peak amplitude, are at least two orders of magnitude far from the thresholds of cardiac fibrillation.

The capability to focus the **E** amplitude distribution in the cerebellar volume (Table 4) does not seem to be linked directly with the age of the child, but with the skull thickness (Figure 6). An increased skull thickness in the occipital bone (Table 1) seems indeed to increase the spread of the electric field within the cerebellum, even toward its anterior part.

Similarly, the increase with age of the CSF volume (Table 1), characterized by higher conductivity than the cerebellum, implies also a greater shunting of the current, which affects the amplitude of the electric field in the cerebellum, lowering both peak and median levels (Figure 6). This finding is in line with the similar results shown on tDCS (Kessler et al., 2013).

### tsDCS

The **E** amplitude induced by spinal tDCS along the spinal cord presents a remarkable increase both in mean and maximum levels with respect to the previous modeling study on adult and adolescents (Parazzini et al., 2014a).

The maximum increase was found at thoracic levels (Figure 8), where the mean **E** level averaged over the four child models, can double the mean **E** level averaged over the three adult/adolescent models, with other conditions being equal. This is probably due to both anatomical and geometrical factors. First, in children, the distance between the active electrode on the back and the spine is lower. This confirms also the higher levels found in the spinal nerves (Figure 9) and the highest levels found in the spinal cord of Thelonious, whose distance between the spinal column and the active electrode is lower than the one of the other models. However, one should consider that these levels are several hundred times below the threshold for neural tissue damage (25 mA/cm^2^ or 14.6 kV/m), but also far from the threshold used in case of invasive spinal cord stimulation (2.3 mA/cm^2^ or 1.35 kV/m; Parazzini et al., 2014b). Second, the minor size of the electrode used here (5 × 3 cm^2^ vs. the 5 × 7.5 cm^2^ in the adults) can better focalize the electric field in the thoracic segment, thus justifying the selective noticeable increase in this segment.

The pattern of the distributions (Figure 7) follows roughly the same behavior in children, in adolescents and adults, with the highest peak and median values of the **E** amplitude distributions (Figures 8, 9) over the thoracic segment, followed by lumbar and cervical segments. Given the different motor functions of each tract, this information can be of basic importance when planning the electrode montage and the modulation current intensity according to the function to be restored.

Moreover, the uniformity of the field (CV lower than 8%; Table 5), as in the previous study, assures that motor and sensory tracts are equally stimulated at the same level. This is slightly higher in lumbar tract probably because of its major thickness than the thoracic tract.

As in case of ctDCS, the median levels of the **E** field distribution along the spinal cord are linked positively with the spinal cord volume and negatively with the CSF volume (Table 1, Figure 10): higher median levels correspond to lower CSF volumes. That is true in the thoracic segment, whereas these correlations are lost in the lumbar tract, where the effects of anatomy probably are felt less in favor of the shape of the tissue and the active electrode distance.

Similarly, the possible dependence of the field homogeneity along the spine with the spinal cord volume in the thoracic segment is due to the fact that in major volumes the field decreases more rapidly than in smaller ones, thus losing in homogeneity (higher CV). In lumbar tract, again, the possible link with the structure (volume) of the spine disappears and in its place the homogeneity is conveyed by the CSF volume and spinal cord length.

As expected with this montage, since the current tends to enter in the active electrode and exit from the reference electrode, the direction of the field in the spinal cord is preferably longitudinal (Table 6). Moreover, an increasing trend with age of the prevalence of the longitudinal component can be clearly identified at thoracic level, due to the increase of the spinal cord length and the consequent increasing distance between the two electrodes.

It is worth noting the prevalence of the longitudinal component along the spine, since there are evidences in literature (Borgens, 1999; McCaig et al., 2005; Hernández-Labrado et al., 2011) that the axonal regrowth is facilitated by longitudinal fields of a slightly higher intensity (5 V/m) than the ones found here.

As to the cardiac safety, the** E** field peaks in the heart here range from 1.6 V/m to 3.6 V/m (Table 7) or, in terms of **J** amplitude, from 0.08 A/m^2^ to 0.19 A/m^2^, and even if higher than the levels induced by ctDCS, they are still considerably lower than the cardiac fibrillation threshold (Reilly, 2012; Parazzini et al., 2013).

As a final remark, in the interpretation of these results, as mentioned above (Figures 1, 2 description), one should take into account the modeling approximation that somehow affects the rigor of the computation. At the head level, there are indeed some sections in which CSF volume is not well segmented and as a consequence, the brain matter and the skull are in contact. That, could change the directional characteristics and amplitude levels at the boundary of the brain matter, where the **E** peaks occur, even if it is already partially accounted by the attempt to link anatomy with electric quantities, as done in this study.

Moreover, the modeling constraints also concern the limited resolution used to segment the spinal structure, represented here as a homogeneous elongated solid of few voxels, without distinguishing between the H-shaped central gray matter, the white matter around it, the blood vessels and, somewhere, the spinal nerves.

The sometimes poor quality of segmentation of spinal nerves and the absence of spinal cord at lumbar and sacral levels or, as in case of Eartha, the absence of spinal nerves at all levels, could indeed affect in particular the directional behavior and the uniformity of **E**. For example, the interruption of Roberta’s spinal cord at lumbar level, which is not completely accounted in the proposed link with anatomy, could create local high levels at the interruption site and modify the median levels.

Therefore, all these factors together could likely change the electric field direction and intensity, and hence it is not to exclude that a more accurate modeling at higher resolution would partially affect the precision of the results.

To conclude, in this study we showed that despite the inter-individual anatomical variability, the electric field induced by ctDCS and tsDCS can reach both the cerebellum and the spinal cord/nerves with amplitude levels ranging between 1 V/m and 14 V/m indicated as the intensities able to modulate the nervous tissue activity (Priori et al., 2014).

The levels found here are slightly higher than the ones previously calculated on adults and adolescents, thus suggesting that the use of tDCS in pediatric age should be conservatively reconsidered on the basis of individual anatomical difference. Roughly, our results suggest an increase of the peak levels of about 40% in the cerebellum and of about 60% in the spinal cord, thus indicating that, in terms of safety, an intensity of 1.2 mA for both techniques, should guarantee the same peak levels found in adults with 2 mA and 3 mA of intensity for ctDCS and tsDCS, respectively. However, we also found that the anatomical variability affects the **E** distribution, both in terms of **E** amplitude and spread/homogeneity, in a complex way, hence it would not be prudent to adjust stimulation dose for children through an arbitrary rule of thumb, but several features have to be considered.

Additionally, one should take into account that the developing brain of a child could react differently than adult to the same amount of applied current. It is indeed known from literature that the tissues’ electric conductivity, which mediates the interaction between current and human tissues, changes with age on the basis of a different tissue structure and composition (Gabriel et al., 2009).

Moreover, children and adolescents show accelerated neuronal plasticity compared to adults (Brunoni et al., 2012). Overproduction of synapses during postnatal development in children contributes to enhanced plasticity by providing an excess of synapses that are pruned during early adolescence. Therefore, brain stimulation techniques, such as tDCS, by enhancing brain plasticity, could interfere and in some case also worsen brain development in an unintended way (Johnston, 2009).

## Author Contributions

PR, AP and MP conceived the design of the study and, together with SF, contributed to the analysis and interpretation of the results. PR and MP supervised the computational simulations. SF ran the computational simulation and drafted the manuscript. All the authors critically revised the draft of the manuscript, approved the submitted version and agree to be accountable for all aspects of the work in ensuring that questions related to the accuracy or integrity of any part of the study are appropriately investigated and resolved.

## Conflict of Interest Statement

AP is stakeholder in Newronika s.r.l., a spin-off company formed by the Fondazione IRCCS Ca’ Granda Ospedale Maggiore Policlinico and Università degli Studi di Milano. All the other authors declare that the research was conducted in the absence of any commercial or financial relationships that could be construed as a potential conflict of interest.

## Footnotes

^1^www.speag.com
